# A Patient-Centered Perioperative Mental Health Intervention Bundle: A Multi- and Mixed-Method Adaptation Study

**DOI:** 10.21203/rs.3.rs-2451723/v1

**Published:** 2023-01-19

**Authors:** Joanna Abraham, Alicia Meng, Ana Baumann-Walker, Katherine Holzer, Emily Lenard, Kenneth E. Freedland, Eric J. Lenze, Michael S. Avidan, Mary C. Politi

**Affiliations:** Washington University in St. Louis

**Keywords:** surgery, perioperative, tailoring, depression, anxiety, geriatric, anesthesia

## Abstract

**Background:**

Anxiety and depression are common among older adults and can intensify during perioperative periods, but few mental health interventions are designed for older surgical patients’ unique needs. We developed and adapted a perioperative mental health (PMH) bundle for older patients comprised of behavioral activation (BA) and medication optimization (MO) to ameliorate anxiety and depressive symptoms before, during, and after cardiac, orthopedic, and oncologic surgery.

**Methods:**

We used mixed-methods including workshop studios with patients, caregivers, clinicians, researchers, and interventionists; intervention refinement and reflection meetings; patient case review meetings; intervention session audio-recordings and documentation forms; and patient and caregiver semi-structured interviews. We used the results to refine our PMH bundle. We used multiple analytical approaches to report the nature of adaptations, including hybrid thematic analysis and content analysis informed by the Framework for Reporting Adaptations and Modifications – Expanded.

**Results:**

Adaptations were categorized by content (intervention components), context (how the intervention is delivered, based on the study, target population, intervention format, intervention delivery mode, study setting, study personnel), training, and evaluation. Of 51 adaptations, 43.1% involved content, 41.2% involved context, and 15.7% involved training and evaluation. Several key adaptations were noted: 1) Intervention content was tailored to patient preferences and needs (e.g., rewording elements to prevent stigmatization of mental health needs; adjusting BA techniques and documentation forms to improve patient buy-in and motivation). 2) Cohort-specific adaptations were recommended based on differing patient needs. 3) Compassion was identified by patients as the most important element.

**Conclusions:**

We identified evidence-based mental health intervention components from other settings and adapted them to the perioperative setting for older adults. Informed by mixed-methods, we created an innovative and pragmatic patient-centered intervention bundle that is acceptable, feasible, and responsive to the needs of older surgical populations. This approach allowed us to identify implementation strategies to improve the reach, scalability, and sustainability of our bundle, and can guide future patient-centered intervention adaptations.

## Background

Approximately 14.4 million inpatient and 12 million major ambulatory surgeries are performed annually in the US [1]. Nearly half of these involve patients over age 65 [2]. Older surgical patients can experience anxiety and depression and have an increased risk of postoperative falls, venous thrombosis, delirium, short-term functional dependence, nausea, and vomiting [3-9]. Furthermore, older age combined with perioperative anxiety, distress, worry, or depression can lead to poor outcomes such as morbidity and mortality, pain, and decreased quality of life [8-15].

Psychotherapeutic interventions, or psychological and behavioral treatments aimed at changing behaviors [16], have been used to help patients manage symptoms of anxiety and depression and maintain overall mental health across the perioperative trajectory. Pre-operative psychological care (e.g., pre-operative assessment, guidance, and family support) and postoperative care (e.g., timely feedback, standardized pain management, and psychological counseling) have improved mental health, including anxiety, hostility, paranoia, depression, and psychosis [17]. Additionally, cognitive behavior therapy (CBT), including psychoeducation and reviewing behavioral goals, have alleviated symptoms of preoperative anxiety and depression and have even aided patients in recovering enough to discharge from the hospital earlier [18, 19].

In addition to psychotherapy, pharmacotherapy interventions and medication-based treatments to address health conditions [20] show promise in reducing postoperative anxiety and pain. Patient-controlled midolazam reduced pre-operative anxiety in middle-aged patients undergoing hysterectomies [21]. Similarly, escitalopram from preanesthetia to day 6 postoperatively relieved symptoms of depression in older patients undergoing knee arthroplasty [22].

However, most psychotherapeutic and pharmacotherapeutic interventions were developed for younger adults and may be less effective for older surgical patients due to differences in risks (e.g., types of social determinants [23]) and symptoms (e.g., frailty, multimorbidity [24, 25]). For example, older patients with depression might not respond to selective serotonin reuptake inhibitors or serotonin-norepinephrine reuptake inhibitors, leading to worse physical and cognitive outcomes, such as increased disability, cognitive decline, and increased risk of dementia [26]. Additionally, biases among older patients against mental health care can present challenges to successful psychotherapy [27]. Current interventions to treat depression and anxiety have primarily been developed and tested for younger adults. It is important to understand the unique needs of older patients for intervention adaptation.

We conducted a multi-stakeholder qualitative interview study with 22 patients over age 65 who underwent major orthopedic, oncologic, or cardiac/thoracic surgeries and 18 perioperative clinicians [28]. Patients reported fear and stress about their surgery, difficulty with medication management, fragmented care transitions, and limited clinician-patient communication. Clinicians reported concerns about restarting psychotropic medications while patients were recovering from surgery without complete and accurate medication lists from patients. Clinicians worried about patients’ limited understanding of their medications and suggested that inpatient social work and psychiatric consultation services could help.

These challenges highlighted the need for a mental health intervention for older surgical patients to alleviate symptoms of anxiety and depression before, during, and after surgery. Both patients and clinicians voiced the need for mitigating patients’ fears and uncertainties across the perioperative care continuum, supporting both behavioral changes and psychiatric medication management, especially during the hospital stay; flexibility to address each patient’s characteristics, contexts, and surgical procedures; and, hiring a dedicated perioperative mental health interventionist (e.g., social worker) to deliver the intervention.

**Figure 1:**
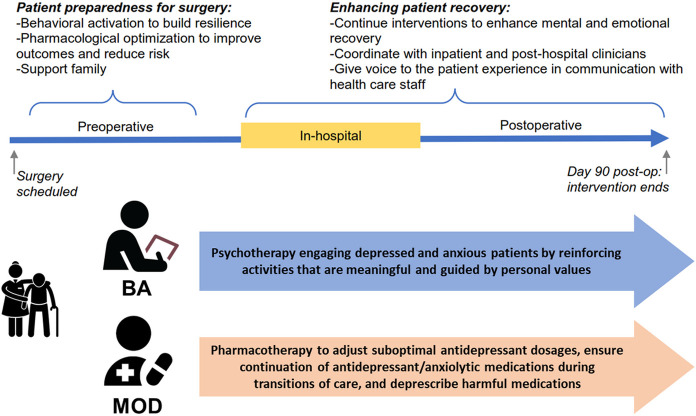
PMH intervention bundle.

Towards this end, we proposed incorporating psychotherapeutic and pharmacotherapeutic treatments into a perioperative mental health (PMH) intervention bundle for older surgical patients [29-31]. Our PMH intervention bundle (Fig. 1) includes behavioral activation (BA), an evidence-based intervention for improving mood by increasing engagement in enjoyable activities [32], and medication optimization and deprescription (MOD) [26, 33]. MOD includes a review and evaluation of patients’ medications to determine if any are eligible for optimization and deprescription. BA and MOD are effective across medically ill populations in several settings and patients [34], especially older patients [35-37]. [38]. MOD can simplify polypharmacy, especially among older patients whose prescription lists grow along with the complexity of their chronic illnesses and whose risk for prescribing cascades increases over time [39].

In this paper, we report on our systematic and multi- and mixed-method, multi-stakeholder tracking and assessment of adaptations to our PMH intervention bundle, in a study running parallel to our feasibility study (NCT05110690) (Table 1). We define adaptation as thoughtful or deliberate modifications made to the intervention to improve its fit within a given context [40].

### Conceptual Framework

**Figure 2:**
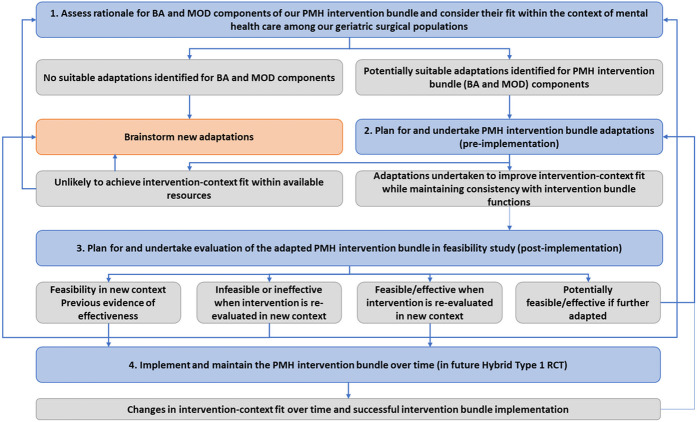
ADAPT guidance for PMH intervention bundle adaptations. Blue boxes indicate stages of step-by-step guidance; grey boxes indicate potential outcomes; directional arrows indicate recommendations for moving between stages.

Our work was guided by ADAPT [42], a framework and step-by-step approach for working with stakeholders; selecting suitable interventions; undertaking and reporting adaptations; and evaluating and implementing these adaptations. Both proactive and reactive adaptations were assessed with stakeholder feedback (Fig. 2).

## Methods

### Study Setting

The study was conducted at a large academic medical center in St. Louis, Missouri, with approximately 18,000 adult inpatient surgeries annually.

### Study Design

Our intervention adaptations were tracked and assessed across two phases: *pre-implementation* (i.e., before the feasibility study) and *post-implementation* (i.e., during the feasibility study). Twenty-four patients from three surgical cohorts (orthopedic, oncological, and cardiac) were enrolled in the feasibility studies. These surgical procedures were selected due to their complex nature and high risk for postoperative complications and other poor outcomes (e.g., delirium, falls [43, 44]). We also documented the fidelity of our bundle [45] (i.e., the degree to which its core components were delivered as intended). During both pre- and post-implementation adaptation phases, we aimed to maintain the core components of the bundle (BA and MOD) while adapting the flexible components based on patient preferences and priorities (see protocol for details on intervention bundle [41]).

Given that this study focuses on describing the intervention bundle adaptation process and the use of multiple methods to track these systematically, it does not include the evaluation results from the feasibility study (see protocol [41]). The Institutional Review Board approved the study at Washington University (IRB#202101103).

### Study Partners and Participants

This study included study partners in both pre-implementation and post-implementation phases, and participants (enrolled patients and caregivers) from the feasibility study in the post-implementation phase only.

### Study partners

In the pre-implementation phase, we organized an internal advisory board (IAB) of study partners from the community and collaborators of the research team The IAB was comprised of patients and caregivers from each targeted surgical specialty; surgeons and nurses from each surgical specialty; community socia workers/interventionists (masters-level clinicians trained in BA and MOD); pharmacists; a health IT administrator; Barnes Jewish Hospital patient experience representatives; and research team members (e.g., treatment developers, informatician). Patients on our IAB have a surgical and mental health history. Patients and caregivers were recruited to the IAB through word of mouth and advertisements at the academic medical center.

### Study participants

In the post-implementation phase, we included patient participants and caregivers from the feasibility study. Patients were at least 60 years of age, scheduled for one of the three identified surgical specialties, with clinically significant depressive or anxiety symptoms (see protocol for further details [41]). Patients’ caregiver(s) were also invited to participate. Caregivers included patient-identified family members or friends who supported their health, safety, and recovery. Patients and caregivers enrolled in the feasibility study were contacted via telephone at the end of the study to gather feedback and suggestions for adaptations.

### Data Collection

Data collection methods, participants, and findings from each method are presented in Table 2.

### Data Analysis

#### IAB workshop studios and periodic intervention reflection meetings

IAB workshop studios, weekly intervention refinement meetings, weekly case review meetings, and periodic intervention reflection meetings were audio-recorded and transcribed.

We performed a hybrid inductive-deductive thematic analysis for each data source [46]. First, an experienced researcher in qualitative methods (JA) read transcripts multiple times for familiarity. JA then openly coded transcripts using data-driven codes (e.g., individuals involved in suggestions, rationale for adaptation) and created an intervention and research adaptation log. Next, JA analyzed adaptations thematically, organizing codes by themes (e.g., intervention bundle component adaptations, study adaptations) and sub-themes (e.g., design and implementation requirements for BA). Discrepancies in analysis were resolved through discussion until 100% consensus was reached with the interventionist team.

#### Weekly intervention refinement meetings and weekly case review meetings

We conducted thematic and content analysis on weekly intervention refinement and case review meetings. Following transcription and review, JA openly coded transcripts using data-driven codes. Then, JA and another researcher (AM) determined coding categories based on the Framework for Reporting Adaptations and Modifications – Expanded (FRAME [40]), a framework to track intervention and implementation strategy adaptations to refine codes based on *what* was being adapted and the nature of the adaptations; *when* did the adaptations happen; *who* suggested the adaptations; and *why* the adaptations were needed. JA and AM coded the content of each meeting in accordance with coding categories (e.g., who includes patients, caregivers, interventionists, etc.) and tallied the frequencies of FRAME-based codes, identifying the most commonly suggested types of adaptations. Table 3 lists a specific example of how we tracked and analyzed adaptations, including sub-theme definitions.

#### Patient and caregiver interviews

Patient and caregiver interviews were audio-recorded and transcribed for hybrid thematic analysis. A qualitative research team (JA and AM or FL) first read transcripts multiple times for familiarity, and then both openly coded transcripts using data-driven codes (e.g., physical challenges, pain, patient perceptions of BA and MO). Second, they identified similar and overlapping codes and factors and categorized them into sub-themes, which were compared within and across transcripts to identify higher-level themes (e.g., barriers to successful BA implementation). These higher-level themes were translated into adaptation suggestions, which were coded using FRAME, similar to how the team analyzed IAB workshop studios, with weekly intervention refinement meetings, case review meetings, and periodic intervention reflection meetings. Discrepancies in coding were resolved through discussion and consensus with the interventionist team.

#### Intervention session audio-recordings and session documentation forms

Audio-recordings of intervention sessions and documentation forms were analyzed for adaptations by our interventionists across patients and for fidelity to the core components of our intervention bundle. Session documentation forms completed by the perioperative wellness team were analyzed using a deductive thematic analysis approach: adaptations noted on these forms were analyzed at the patient level and then higher-level themes on adaptations across patients were organized using FRAME.

Second, the intervention bundle fidelity was assessed using a structured fidelity rating checklist **(Appendix S1)** developed by our interventionist team and evaluated for language and clarity by researchers (JA and KF). The fidelity rating checklist mirrored the core components of the intervention bundle. After piloting the rating checklist, an undergraduate researcher (trained by JA) listened to the audio-recordings and documented how well the interventionists were delivering the intervention bundle as intended in the standard operating procedures (SOPs) or deviating from the intervention bundle SOPs. A PhD-level social worker (KH) randomly selected 20% of these intervention session recordings and completed the fidelity rating checklist. Fidelity was rated based on four core components: personalized rationale; values and goals assessment; activity scheduling; and activity tracking. Personalized rationale and values and goals assessment were considered completed if were discussed with the participant during at least one BA session. Activity scheduling and tracking were considered complete if participants engaged in scheduling activities during at least 80% of the BA sessions after session 3 (out of a maximum of 10). The IRR was calculated between the two researchers for all items on the fidelity rating checklist (Cohen’s k = 0.76, consistent with a high level of agreement).

## Results

Data were systematically collected between June 2021 and September 2022. 29 IAB study partners (including 15 patients and caregivers) and 19 patients and caregivers from our feasibility study participated in our adaptation assessment (Table 4).

Table 5 displays our adaptation evaluation findings, divided into pre-implementation and post-implementation themes and data. For example, one adaptation involved simplifying BA activity forms – this adaptation was coded as a pre-implementation content adaptation that involved tailoring, rewording, or refining. As a planned adaptation at the target intervention group level, we confirmed that the adaptation adhered to the core components of the intervention bundle and served to increase its effectiveness.

Out of 51 adaptations, content adaptations represented 43.1% (n = 22), contextual adaptations 41.2% (n = 21), and training and evaluation adaptations 15.7% (n = 8). The most common nature of adaptations was tailoring/rewording/refining adaptations (n = 18, 35.3%), while the most common level of adaptation was at the individual patient or practitioner-level (n = 37, 72.5%). Additionally, most adaptations were based on practical considerations (n = 26, 51.0%), were reactive (n = 28, 54.9%), and served to increase implementation or the ability of staff to deliver the intervention bundle successfully (n = 21, 41.2%). In the sections below, we describe these adaptations across pre- and post-implementation phases.

### Content Adaptations

We identified 22 content adaptations (n = 22/51; 43.1%): 9 were pre-implementation (17.6%), and 13 were post-implementation (25.5%) (Table 6).

### Pre-implementation adaptations

One example of a content-based adaptation includes renaming interventionists. During IAB Studio #2, study partners discussed intervention language and expressed that the term “perioperative wellness partner (PWP)” could better reflect the intervention bundle deliverer’s holistic training while emphasizing a comfortable environment for patients to improve their wellness after surgery. Other IAB members agreed, stressing that someone trained to speak reassuringly with patients and serve as a mental health advocate was necessary, as the patient would rely on the bond formed throughout the entire perioperative process. In a similar discussion about language and patient acceptability, the term “medication optimization and de-prescription” was refined to “medication optimization (MO).” This phrase felt less intimidating to patients, who were previously wary about stopping any of the medications they already took. This planned adaptation occurred at the target intervention group level and was intended to align the intervention bundle with organization goals better.

In another example, across IAB Studios #2 and #3, several IAB members suggested that it could be difficult for patients to connect with and trust strangers with personal issues during their first session, especially over the phone. One PWP also emphasized that there was a *“need for rapport building [first], so that we actually can personalize it. It's kind of… hard to personalize it when you don't know the patient that well and you're kind of working to get to know them through that.”* Thus, building a relationship with the PWP was crucial and was recommended prior to beginning BA. Following further discussion, the research team decided to modify the content of the first session to focus on building trust and rapport and introducing the patient to the intervention and its core components (e.g., personalized rationale). This planned adaptation occurred at the individual patient/practitioner level and was intended to increase the effectiveness of the intervention bundle.

### Post-implementation adaptations

During a periodic intervention reflection meeting, PWPs noted that patients had difficulty following the activity scheduling and tracking documentation forms that they were assigned. One PWP, for example, stated, *“Having [patients] strictly write stuff down… they don't really seem to need that.”* Similarly, Cardiac-Patient-3’s documentation form noted that they did not track or schedule activities according to their PWP’s instructions but remained very active and talked to their PWP about their recovery period activities. Similarly, Orthopedic-Patient-2 voiced that they *“had the same problem when [they] went through trauma therapy. [They] just don't write things down.”* Orthopedic-Patient-60 declined to log their activities, so their PWP proposed that they review their calendar at each session and recall activities without writing them down. Thus, BA documentation forms were reduced in detail and emphasized meeting the patient where they were, suggesting but not requiring activity documentation, with PWPs encouraged to offer flexible methods of activity documentation (e.g., journaling). Loosening the structure of the BA documentation form was a reactive adaptation that occurred at the individual patient/practitioner level to increase patient reach, participation, and access to the intervention bundle.

### Contextual Adaptations

We identified 21 contextual adaptations (n = 21/51; 41.2%): 6 were pre-implementation (11.8%) and 15 were post-implementation (29.4%) (Table 7).

### Pre-implementation adaptations

One significant pre-implementation contextual adaptation involved forming separate teams for each intervention bundle component, as feedback indicated that the PWP would require real-time assistance during MO to correctly identify which medication changes could benefit the patient. At IAB Studios #2 and #3, patients and caregivers remarked that they wanted to see clinicians handling their medications directly rather than through consultation with ancillary staff and researchers. Several clinicians for leading MO were suggested, including primary care physicians and pharmacists. Ultimately, the research team established that the new surgical wellness program would comprise two teams: the PWP team and the pharmacy team. The PWP team managed BA and supervised all sessions, while the pharmacy team (pharmacists and a geriatric psychiatrist) managed MO. This planned addition to the intervention bundle occurred at the target intervention group level and served to increase the ability of staff to deliver MO successfully.

### Post-implementation adaptations

Across periodic intervention reflection meetings and interviews, PWPs and patients noted that their overall success with the intervention bundle relied heavily on building trust and warm relationships during sessions. Cardiac-Patient-1 noted that their PWP was *“very sensitive… Very caring.“* They elaborated, *“I feel with all my heart that [my PWP] really helped me through a tough time… and cared about… my health and my well-being.”* Similarly, Oncologic-Patient-5 said, *“[My PWP] wasn’t judgmental. [They were] totally understanding.”* This also aligned with findings from weekly patient case review meetings, as other PWPs agreed that including elements of emotional validation and warmth was important for intervention bundle success. Thus, to maintain the standard of empathetic care, the research team opted to incorporate elements of compassion into the intervention bundle protocol, adapting each session to be more interactive and patient-sensitive. PWPs were instructed to reassure patients that their sessions were flexible and personalized for their preferences, schedules, and needs. Adding these elements to the intervention bundle was a planned adaptation at the target intervention group level and served to align the intervention bundle with organization goals.

Additionally, patients suggested that during recruitment, before scheduling sessions, PWPs should provide more detailed explanations of the intervention bundle using more straightforward language. Cardiac-Patient-2 explained, *“[Details about the intervention bundle] should be told to people before the surgery and they need to know what benefits are [and] what to do. And who to talk to for help.”* Orthopedic-Patient-2 and others felt that they went into the study not fully understanding what they needed to do and how the intervention bundle would help them, and only realized partway through the study. This was also evident in our session documentation form analysis: some patients did not understand what they were supposed to do before study participation and were ultimately not interested in the intervention bundle upon finding out more details throughout sessions. For example, Orthopedic-Patient-6’s documentation form on their 5th session indicated that *“the patient opted to withdraw from the study. [They] stated that this is something that doesn’t interest [them],”* after two missed sessions and two sessions where they declined to complete the BA instructions. Thus, the research team modified consenting language to include a more thorough description of what to expect from the intervention bundle. This reactive adaptation on an individual patient/practitioner level served to increase reach, participation, and access to the study for patients.

Furthermore, we noted three key differences between surgical cohorts throughout the feasibility study: pre-operative timelines, session schedules, and patient needs. First, we observed substantial differences among the cohorts in perioperative timelines. Orthopedic patients typically scheduled their surgeries over 3 months in advance, oncologic patients scheduled their surgeries about 2 weeks in advance, and cardiac patients scheduled their surgeries about 2–3 days in advance. Therefore, orthopedic patients typically had more time preoperatively to start the intervention and plan pre-operative sessions, while cardiac patients had little pre-operative preparation time. This was noted for future implementation considerations to better shape intervention plans for each cohort.

Second, differences in postoperative schedules and medical treatment were observed between cohorts, resulting in changes to session frequency. For example, oncologic patients often required continued chemotherapy and thus could not attend BA sessions as frequently; they needed sessions every 2–3 weeks (vs. 1–2 weeks). Similarly, orthopedic patients were often busy with physical therapy following surgery, resulting in sessions scheduled every 2–3 weeks.

Third, each cohort had specific surgery-based needs and priorities and utilized different activities and techniques. For example, oncologic patients often had trouble sleeping due to extensive discomfort and had difficulties with physical recovery. As such, PWPs adapted their recommendations to provide sleep hygiene suggestions. Oncologic-Patient-2 explained that their PWP aided them in sleep hygiene strategies and felt that the BA components helped them with *“incorporating [techniques] into the evening and the morning routine,”* which benefitted them. In another example, orthopedic patients typically had a physical therapist and received exercise instructions to strengthen replacement joints. Thus, PWPs established physical goals more frequently for them.

### Training and Evaluation Adaptations

We identified 1 pre-implementation (n = 1/51; 2.0%) and 7 post-implementation training and evaluation adaptations (n = 7/51; 13.7%) (Table 8).

### Pre-implementation adaptations

Before intervention bundle implementation, study partners agreed during IAB Studios #1, #2, and #3 that training interventionists were essential to obtain patient buy-in and trust in the intervention bundle. Patients were apprehensive about social workers, since they were skeptical about the potential lack of intervention training or experience in delivering mental health interventions. Thus, all PWPs recruited for this study came with training and prior experience in mental health. They were also trained in BA by Puspitasari and colleagues (using BASA training modules) [47]. Training sessions consisted of four 1-hour weekly sessions covering 4 core BA strategies and oriented the trainers to BA SOP content and resources. In addition to discussing core BA strategies, PWPs were taught how to model BA for patients, lead BA sessions, and provide feedback.

Weekly intervention meetings also helped PWP and pharmacy team to review the SOPs and materials with BA and MO treatment developers and receive continuous feedback. This planned addition to the study was conducted at an individual practitioner level and increased the ability of staff to deliver the intervention bundle successfully.

### Post-implementation adaptations

Several training sessions were incorporated across intervention implementation as both refreshers and to introduce new elements to the study. First, intervention lead coordinators (EL and KH) provided several refresher training sessions and materials to train all PWPs on introducing the study, introducing the intervention bundle, and working through each form with patients. Training sessions were held over four 1–2 hour video conference meetings and included a mix of didactic and interactive content (e.g., role-playing). The sessions provided an orientation to the revised intervention manual and the objectives for each session, along with a review of unchanged core components and instructions on future work adaptations. This added element was planned at an individual practitioner level and was based on practical considerations to increase staff’s ability for successful intervention delivery.

Another post-implementation adaptation to training involved adding pharmacy students to the pharmacy team to provide further expertise regarding medication optimization and education for patients. Training sessions were led by the two study team clinical pharmacy specialists with multiple weekly sessions. Session content included good clinical practices; review of the MO SOPs; electronic health record access and navigation; intervention database navigation; and demonstrating compassion and empathy during patient communication. Students were given supplemental readings about antidepressant dosing and potentially harmful medications. This reactive addition to study training occurred at an individual practitioner level and was based on practical considerations to increase staff’s ability for successful intervention delivery.

In addition, several evaluation adaptations were made to the study as reactive responses to data and outcome collection difficulties. For example, patients were evaluated originally at 1-month, 2-month, and 3-month follow-ups. However, the 2-month follow-up was removed from the SOP due to difficulty in following up with patients. This reactive removal of an element occurred at the target intervention group level and served to increase reach, participation, and access to the study.

### Intervention Fidelity

Personalized rationale was discussed for 87% of patients (n = 20), and values and goals assessment were discussed for 78% of patients (n = 18). Only 26% of participants engaged in activity scheduling (n = 6) and 17% in activity tracking (n = 4) for at least 80% of sessions after session 3.

A few patients (especially those who withdrew from the study) did not follow the core components of the intervention bundle. For example, Oncologic-Patient-2 was uninterested in BA and MO, and their sessions mainly consisted of their PWPs checking in on their recovery and activities. This content-based removal of BA and MO elements increased participation in the study but lacked fidelity to the original intervention bundle’s purpose and functions.

However, most patients followed the core components of the intervention bundle while altering or skipping some BA and MO steps. For instance, Cardiac-Patient-5 did not schedule any activities, but was very motivated to recover from their procedure, so their PWP worked with them to identify goals, priorities, and strategies for meeting them. This refining of the BA SOP let the patient decide how to utilize BA in a way that worked for them. Even though they did not schedule activities, they planned to do them on their own terms throughout the week, increasing the effectiveness of the intervention bundle and adhering to its components.

## Discussion

We reported on the systematic adaptation process of a PMH intervention bundle for older adults to ensure intervention bundle feasibility and sustainability across three different surgical settings.

### Use of Implementation Science Methods and Implications

Our comprehensive and robust approach to adaptation led to the development and refinement of our PMH intervention bundle that we anticipate will be acceptable, appropriate, and feasible for patients and PWPs in our full trial. Using the ADAPT framework to capture process [42], and FRAME to capture adaptations [40], our multi-pronged, multi-method approach identified methods to develop an adaptable intervention bundle for older patients in the perioperative context in three different cohorts (orthopedic, cardiovascular, and cancer patients).

Our data revealed differences in the types of adaptation between the pre-implementation and the post-implementation phases. In the pre-implementation phase, we reported more planned adaptations, mostly around content, to fit the intervention to the perioperative setting. In the post-implementation phase, the number of reactive content and contextual adaptations increased. Several elements (e.g., adding compassionate modules) were added in the post-implementation phase. These data indicate the importance of feasibility trials to develop adaptable interventions to increase the probability of fitting evidence-based interventions in new settings and/or for new populations. Specifically, pre-implementation adaptation work has historically supported intervention-context fit and has contributed to intervention sustainability [48]. Spending time adapting the bundle to increase the fit with the contexts hopefully will increase the probability of success and future sustainment of the intervention.

One of the main challenges in the field of adaptation is examining *how* to adapt and track adaptations along the process [40] as the literature has scant examples of adaptations done in different phases of implementation [49]. We used multiple methods to triangulate the needs of patients and PWP with feedback from the advisory board and research team – this allowed us to ensure the fit of the bundle in the three settings [50]. While our approach was time-consuming and exhaustive with multiple iterations of feedback, discussion, and adaptation over the feasibility study and the planning period before implementation, it provided a robust understanding of needed adaptations. However, others may find our approach not feasible and may employ a simplified version of our methods. Nevertheless, this adaptation assessment and tracking process can guide future patient-centered intervention adaptations while ensuring that they remain consistent with the original design and goals.

### Adapted PMH Intervention Bundle

**Figure 3:**
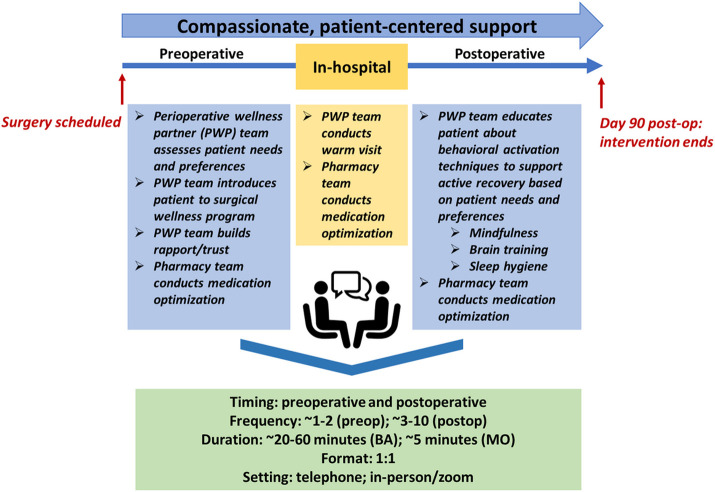
Adapted perioperative mental health intervention bundle.

Our PMH intervention bundle (Figure 3), dubbed the “surgical wellness program,” is pragmatic and collaborative, with both reproducible, generalizable core components (e.g., a dedicated pharmacy team, simplified BA documentation forms, and emphasis on compassionate care) and adapted, patient-driven components (e.g., varied activity scheduling and tracking methods and surgery-specific preferences for BA activities). We describe significant adaptations below, with details of the intervention bundle according to the TIDieR checklist **(Appendix S2)** [51].

First, activity BA scheduling and tracking forms are now flexible and based on patients’ comfort and preference in entering necessary information; for example, some patients may prefer writing their activities down in a journal, while others might note their activities in their mobile phone. Second, BA activities now depend heavily on each individual patient’s preferences and surgical recovery. Furthermore, PWPs demonstrate flexibility in scheduling and session agenda planning according to each patient’s mood during sessions. Examples of supplemental behavioral activities include activity plans for self-directed mindfulness practice, sleep hygiene exercises, and evidence-based cognitive training.

Third, surgery-based protocols are adjusted according to the different priorities, pre-operative timelines, session schedules, and patient needs of each surgical cohort. For example, orthopedic patients can schedule more pre-operative sessions, while oncologic patients can schedule one or two sessions before their surgeries. Cardiac patients can either schedule one session prior to surgery or do postoperative sessions only.

Fourth, our PMH intervention bundle takes on a shared decision-making approach with patients and is flexible for each patient’s needs and types of surgeries. Our data found the importance of the PWP establishing trust with their patient and assessing patient needs and preferences while approaching their situation with empathy and compassion. Our data also showed the importance of giving the patient the option of using MO to reduce problematic medications and increase sub-therapeutic doses of antidepressant medications to therapeutic levels through a collaborative approach. In the immediate postoperative period, giving the option for the PWP and pharmacy team to visit the patient in the hospital is important. Following discharge, patients can choose how to utilize BA activity scheduling and tracking to support an individualized, active recovery. Patients can also schedule more or fewer sessions depending on their physical recovery progress and level of stress.

In the updated PMH intervention bundle, two main components are integrated to prepare cardiac, oncologic, and orthopedic patients for surgery and to promote enhanced recovery. MO reduces the use of problematic medications and increase sub-therapeutic doses of antidepressant medications to therapeutic levels. Through a collaborative approach, medication adjustments are made with the patient and pharmacy team. Additionally, BA engages patients in activities that are personally rewarding, supporting an individualized, active recovery from surgery, and encouraging patients to gradually re-engage in the important activities and cope with perioperative stress.

The PMH intervention bundle is carried out remotely via phone or secure web conference, with optional in-person visit/s while the patient is hospitalized. Each patient is assigned a PWP, who works with the patient prior to surgery and approximately 3 months after surgery. Preoperatively, the PWP establishes trust with their patient, assessing patient needs and preferences while approaching their situation with compassion. Meanwhile, the pharmacy team assesses patient medications and discusses recommendations with the patient and inpatient care teams, ensuring any changes are maintained in-house following surgery. Within the hospital, the pharmacy team conducts MO again if any further changes needed to be made. Finally, at home post-discharge, wellness partners work with patients on BA (e.g., activity planning, activity logging). Further MO guidance can be provided at the patient’s request.

One-on-one sessions occur on a weekly basis initially and then are reduced to every two weeks or according to the patient’s needs and preferences for a total of up to 10 sessions. Session duration is approximately 40 minutes per session but can be adjusted depending on patient needs and treatment goals.

All adaptations to the intervention bundle ensure personalized perioperative mental health care delivery. Although we have made several changes, we maintain the core components of the original intervention bundle and ensure its fidelity. In other words, the underlying functions of targeting behavioral change and medication optimization remain constant, preparing older patients for surgery through BA to build resilience and through MO to improve medication regimens and reduce risk of complications.

### Future Work

We will conduct a Hybrid Type 1 Effectiveness-Implementation RCT to assess the effectiveness and implementation-potential of our adapted PMH intervention bundle in 300 older surgical patients across cardiac, orthopedic, and oncologic cohorts. Our control condition will receive printed mental health resources from our team, while the intervention condition will receive our PMH bundle. Our primary outcome of interest is depression/anxiety. Exploratory outcomes include quality of life, medication list, delirium, length of stay, rehospitalization, falls, PMH intervention bundle reach, implementation potential (acceptability, appropriateness, feasibility), and overall patient experience.

### Limitations

We acknowledge study limitations. *First*, the approach was resource-intensive, requiring iterative data collection, analysis, and integration from several sources and stakeholders. Nevertheless, this can also be considered as a strength of the approach as it allowed us to conduct a thorough examination of necessary adaptations to suit our target surgical population’s needs, priorities, and preferences, thereby improving the rigor in our intervention adaptation process. *Second*, our adaptations to the bundle were informed by the needs of our participants and advisory board members who may not be representative of diverse backgrounds (e.g., racial/ cultural differences). However, we are currently conducting a follow-up study to investigate this particular aspect, which is supported by our Diversity, Equity, and Inclusion Community Advisory Board. *Third*, given that this is a single-site study, feedback gathered might not have been representative of the overall target population’s needs and preferences; additionally, results may not be generalizable from academic to community hospital settings. We plan for future multi-site evaluation that will help us refine the bundle to meet the needs of patients across both rural and urban settings.

## Conclusions

Mental health symptoms are a significant issue in the perioperative setting and can worsen adverse surgical outcomes. Across the literature, several studies have reported on interventions to address perioperative depression and anxiety, but often for general adult surgical populations, not specifically older adults. Additionally, few studies have utilized mental health interventions along the entire perioperative timeline from pre-operative preparation to postoperative at-home recovery. In response to a pressing need for perioperative mental health interventions adapted for an older surgical population, we identified evidence-based mental health intervention components from other settings and adapted them to the perioperative setting for older adults in a novel study. Tracking and assessing adaptations through multiple methods, we have created a pragmatic and patient-centered intervention bundle comprised of BA and MO components that fits the needs of older surgical populations.

## Figures and Tables

**Table 1:** Details on adapted perioperative mental health bundle components ([41]).

Intervention Bundle	Behavioral Activation	Medication Optimization and Deprescription
Interventionist	Trained social worker	Trained social worker and pharmacy team consisting of pharmacists and a geriatric psychiatrist
Description	Behavioral psychotherapy that helps depressed and anxious patients through identifying and tracking enjoyable and meaningful activities guided by personal goals and priorities [52].	Pharmacotherapy that helps to adjust suboptimal psychotropic dosages, deprescribe unnecessary or harmful medications, and ensure psychotropic continuation across the perioperative period [53, 54].
Core active components	• Personalized Rationale Identification • Values and Goals Assessment • Activity Scheduling • Activity Monitoring	• Reconcile patient’s medications • Identify the patient’s likely need for, and interest in, a medication adjustment • Make medication adjustments • Assess the response to that adjustment • Coordinate with hospital team to ensure medication changes introduced preoperatively are maintained in-house • Ensure medication changes are reconciled during transitions of care
Adapted, patient-driven components	• Selected behavioral activation activities: depending on patient needs and preferences • Timing: Pre-operative and postoperative phases • Format: 1:1 (patient-specific activities); group sessions (to share experiences with others and hear about other stories – peer-motivation) • Duration: 20–60 minutes • Frequency: 1–4 (presurgery); 2–12 (postsurgery) • Setting: In-person (first time – surgeon clinic/preop counseling class) and zoom (video)	• Timing: Pre-operative and postoperative phases (start as early as possible) • In-hospital care: Pharmacy team coordinates with in-hospital team to ensure continuity of care • Format: 1:1 session in-person/telephone/online • Duration: 5 min • Frequency: 1–4 (pre); 2–12 (post) • Setting: In-person (first time); zoom (video) or in-person (for remaining sessions)

**Table 2:** Data collection methods. Pre-implementation refers to time-period prior to intervention implementation before the feasibility study; post-implementation refers to time-period after intervention implementation during and after the feasibility study.

Purpose of Method	Phase	Participants	#	Findings
**IAB workshop studios**: To obtain different stakeholders’ perspectives and experiences: patient mental health needs, intervention objectives, and adaptations required for interventions content and delivery, as well as study delivery.	Pre-implementation	IAB study partners (Studios 1 and 3: patients and caregivers; Studio 2: all IAB members)	3	We identified barriers and facilitators to intervention implementation based on study partner perspectives and brainstormed adaptations to make.
**Weekly Intervention refinement meetings**: To identify pre-implementation adaptations necessary for successful PMH intervention bundle use among interventionists and patients.	Pre-implementation	Interventionists, social workers, pharmacists, psychiatrists, behavioral scientists, and research team members	12	We assessed progress in intervention bundle design We identified pre-implementation barriers to intervention delivery and brainstormed adaptations to make accordingly.
**Periodic intervention reflection meeting**: To reflect upon interventionists’ experiences, to collect contextual data and triangulate data for a richer understanding [49].	Post-implementation (mid-point)	Implementation scientists, interventionists, interventionist supervisor	1	We assessed study progress and interventionist experiences. We also identified barriers to intervention delivery and brainstormed adaptations to make accordingly.
**Weekly patient case review meetings**: To review and discuss patient intervention sessions and to document adaptations and challenges to intervention implementation.	Post-implementation	Interventionists, social workers, pharmacists, psychiatrists, behavioral scientists, and research team members	33	We assessed study progress and intervention bundle use among patients. We also identified post-implementation barriers to intervention delivery and brainstormed adaptations to make accordingly.
**Audio-recordings of intervention sessions and collection of session documentation forms completed by interventionists**: To capture data on progress towards MOD (adherence to medication changes, side effects) and BA (goals, values, activity scheduling and assessment) components; to also assess intervention fidelity through intervention delivery (delivering PMH intervention bundle consistently), intervention receipt (reflection of patients’ receipt and understanding of the PMH intervention bundle and their capacity to use skills taught), and intervention enactment (patients’ actual performance of MOD and BA skills and implementation of core intervention components) [55, 56].	Post-implementation	Patients and interventionists	226	We assessed intervention fidelity to core components of MOD and BA and recorded any adaptations made during each session.
**Patient interviews and caregiver interviews**: To assess perspectives on the intervention and study overall.	Post-implementation	Patients and caregivers from feasibility study	19	We identified patient suggestions for future improvement to study content and implementation (for adaptation evaluation only).

**Table 3:** FRAME definitions and example of tracking document.

FRAME categories	Sub-categories	Example
Date of adaptation	When was the change made?	8/25/2021
**Description of adaptation**	What has been changed?	BA session documentation forms were revised to be different for Sessions 1, 2–9, and 10.
**WHAT is adapted?**	*Content*: changes made to content itself, or that impact how aspects of the treatment are delivered *Contextual*: changes made to how the intervention is delivered, based on the study/research, target population, intervention format, intervention delivery mode, study setting, or study personnel *Training and evaluation*: changes made to how staff are trained or how the intervention is evaluated	Contextual: format
**What is the NATURE of the intervention adaptation?**	How did the intervention, study, or training and evaluation change? *Tailoring/rewording/refining*: a change to the intervention that leaves all of the major intervention principles and techniques intact *Integrating intervention into another framework*: another treatment approach is the starting point, but elements of the intervention are brought into the treatment *Integrating another treatment into the intervention*: the intervention is the starting point, but aspects of different therapeutic approaches or evidence-based practices are also used *Removing/skipping elements*: intervention baseline or standard treatment is based on the evidence-based practice, but particular elements are dropped *Lengthening/extending (pacing/timing)*: a longer amount of time than prescribed by the manual is spent to complete the intervention or intervention sessions *Adjusting the order of intervention components*: intervention modules or concepts are presented in a different order than originally described in the manual *Adding elements*: additional distinct materials or areas of focus consistent with the fundamentals of the intervention are inserted *Departing from intervention (drift)*: use of another intervention *Loosening structure*: the structure of intervention sessions is different from what is prescribed in the manual, but the code is appropriate *Repeating components*: a module or intervention that is normally prescribed once during a protocol is done more than once *Substituting components*: a module or activity is replaced with something that is different in substance	Tailoring/rewording/refining
**Was the adaptation proactive or reactive?**	*Proactive- Planned*: Part of the plan to modify to maximize fit and implementation success *Reactive- Unplanned*: often in response to an obstacle, challenge, deviation from the plan	Proactive
**At what LEVEL of DELIVERY is the content level adaptation?**	For whom does the modification apply? *Individual patient/practitioner level*: individuals who make the adaptation for their own involvement in the study *Target intervention group level*: all individuals with the problem that is being targeted *Clinic/unit-level*: an entire unit or clinic makes an adaptation *Hospital level*: the full organization makes the adaptation *System level*: the healthcare system, county, or community makes an adaptation	Target intervention group level
**HOW or on what basis was this change made?**	*Based on vision or values* *Based on a framework* *Based on knowledge and experience working with patients* *Based on practical considerations* *Based on financial incentives/payments* *Based on feedback or suggestions*	Based on practical considerations
**WHY? What is the purpose of the adaptation?**	What is the intent or goal of the adaptation? *Increase reach, participation, access* *Increase effectiveness* *Increase adoption by more settings* *Make intervention more aligned with organization goals* *Increase implementation/ability of staff to deliver intervention successfully*	Increase implementation/ability of staff to deliver intervention successfully
**WHO suggested the decision to adapt?**	Who suggested the decision to adapt? *Interventionists* *Pharmacists* *Health IT administrator* *Research team members* *Patients* *Caregivers*	Interventionists Research team members

**Table 4:** Study partner and participant demographics. *Caregivers were not asked to respond with race and ethnicity for the study.

IAB study partners	
Participant type	
Clinicians	
Anesthesiologist	N=1
Social worker	N=2
Pharmacist	N=1
Psychiatrists	N=1
Behavioral scientists	N=1
Registered Nurse	N=2
Surgeon	N=1
Researchers	
Implementation scientists	N=3
Informatician	N=1
Research coordinator	N=2
Patient experience leader	N=1
Patients	
Orthopedic patients	N=3
Oncologic patients	N=2
Cardiac patients	N=2
Caregivers	
Orthopedic caregivers	N=2
Oncologic caregivers	N=2
Cardiac caregivers	N=2
Sex	
Male	N=13
Female	N=16
Race	
White	N=27
Black	N=1
American Indian or Alaska Native	N=0
Asian	N=1
Native Hawaiian or Other Pacific Islander	N=0
Ethnicity	
Non-Hispanic or Latinx	N=28
Hispanic or Latinx	N=1
Feasibility study participants	
Participant type	
Orthopedic patients	N=5
Oncologic patients	N=5
Cardiac patients	N=4
Orthopedic caregivers	N=0
Oncologic caregivers	N=4
Cardiac caregivers	N=1
Sex	
Male	N=9
Female	N=10
Race*	
White	N=12
Black	N=2
American Indian or Alaska Native	N=0
Asian	N=0
Native Hawaiian or Other Pacific Islander	N=0
Mixed	N=0
Ethnicity*	
Non-Hispanic or Latinx	N=13
Hispanic or Latinx	N=0
Prefer not to answer	N=1

**Table 5:** Adaptations identified across implementation timepoints.

Adaptation constructs	Adaptation elements	Pre- implementation	Post- implementation	Total
**WHAT is adapted?**	Content: intervention elements	9	13	22
	Contextual: research, population, format, delivery mode, setting, and personnel	6	15	21
	Training and evaluation: how staff are trained and how intervention is evaluated (e.g., outcomes)	1	7	8
**What is the NATURE of the intervention adaptation?**	Tailoring/rewording/refining	8	10	18
	Integrating another treatment into the intervention	0	1	1
	Removing/skipping elements	1	3	4
	Lengthening/extending (pacing/timing)	0	1	1
	Adjusting the order of intervention components	2	2	4
	Adding elements	6	13	19
	Loosening structure	0	2	2
	Substituting components	0	2	2
**Was the adaptation planned or reactive?**	Planned	16	12	28
	Reactive	0	23	23
**At what LEVEL of DELIVERY is the content level adaptation?**	Individual patient or practitioner level	10	27	37
	Target intervention group level	8	6	14
**HOW or on what basis was this change made?**	Based on vision or values	3	6	9
	Based on a framework	4	1	5
	Based on knowledge and experience working with patients	0	9	9
	Based on practical considerations	8	18	26
	Based on financial incentives/payments	0	0	0
	Based on feedback or suggestions	0	1	1
**WHY? What is the purpose of the adaptation?**	Increase reach, participation, access	3	12	15
	Increase effectiveness	6	4	10
	Make intervention more aligned with organization goals	1	4	5
	Increase implementation/ability of staff to deliver intervention successfully	6	15	21

**Table 6:** Content adaptations.

Adaptations	Original protocol	What was adapted	When adaptation occurred	Planned or reactive	At what level of delivery	Intent of adaptation
Interventionists were renamed to "perioperative wellness partners" or "wellness partners" to use patient-friendly language that accurately and positively describes the clinician-patient relationship, and medication optimization and deprescription was renamed to “medication optimization.”	Originally, study personnel who were trained to deliver the intervention bundle to patients were called “interventionists.” The pharmacotherapy component was originally called “medication optimization and deprescription.”	Tailoring/rewording/refining	Pre-implementation	Planned: Part of the plan to modify to maximize fit and implementation success	Individual patient/practitioner level	To make intervention more aligned with organization goals
Specific mental health-based BA and MO needs, expectations, and goals were identified.	BA was not tailored specifically towards patient mental health needs.	Tailoring/rewording/refining	Pre-implementation	Planned: Part of the plan to modify to maximize fit and implementation success	Individual patient/practitioner level	To increase effectiveness
Wellness partners served as liaisons for mental health support, referring patients to other resources, social work referrals, and financial aid when necessary.	Original protocols gave wellness partners more responsibility over social work and other resources.	Removing/skipping elements	Pre-implementation	Planned: Part of the plan to modify to maximize fit and implementation success	Individual patient/practitioner level	To increase implementation/ability of staff to deliver intervention successfully
MO SOP was revised to focus on pre-operative psych medications and postoperative psych medication changes (including name, dose, units, frequency, start date and stop date, indication).	The original MO SOP focused on all medications.	Tailoring/rewording/refining	Pre-implementation	Planned: Part of the plan to modify to maximize fit and implementation success	Target intervention group level	To increase effectiveness
MO SOP was revised to assess potential for stopping muscle relaxants preoperatively and reflect the difference between PRN/OTC and other prescribed medications.	The original MO SOP did not differentiate between specific medications that did not pertain to intervention bundle goals.	Tailoring/rewording/refining	Pre-implementation	Planned: Part of the plan to modify to maximize fit and implementation success	Target intervention group level	To increase effectiveness
The first session of BA was focused on building trust and rapport and introducing the patient to the intervention and its core components (e.g., personalized rationale). Activity scheduling followed in the next sessions.	Previously, the first session of BA began therapy and goal-setting exercises immediately.	Adjusting the order of intervention components	Pre-implementation	Planned: Part of the plan to modify to maximize fit and implementation success	Individual patient/practitioner level	To increase effectiveness
BA forms included simple activity planning).	BA documentation forms were originally more complex and harder to use.	Tailoring/rewording/refining	Pre-implementation	Planned: Part of the plan to modify to maximize fit and implementation success	Individual patient/practitioner level	To increase implementation/ability of staff to deliver intervention successfully
Wellness partners made medication adjustments and assessed the responses to each adjustment.	Wellness partners originally did not need to check for side effects and responses to medication adjustments.	Adding elements	Pre-implementation	Planned: Part of the plan to modify to maximize fit and implementation success	Target intervention group level	To increase implementation/ability of staff to deliver intervention successfully
Wellness partners coordinated with the hospital team to ensure that medication changes introduced preoperatively were maintained in-house.	No check-ins were originally conducted to ensure continuity of care and medication use in-house.	Adding elements	Pre-implementation	Planned: Part of the plan to modify to maximize fit and implementation success	Target intervention group level	To increase effectiveness
SOPs and documentation forms were revised to use simpler, layman terms for patients to understand.	SOPs originally had too much complex language that was hard for patients to understand.	Tailoring/rewording/refining	Post-implementation	Reactive: Unplanned often in response to an obstacle, challenge, deviation from the plan	Individual patient/practitioner level	To increase implementation/ability of staff to deliver intervention successfully
BA SOP was revised to create tailored sessions (timing, frequency, referrals, resources, etc.).	The BA SOP originally was not tailored to each patient’s personal preference for timing, frequency, etc.	Tailoring/rewording/refining	Post-implementation	Reactive: Unplanned often in response to an obstacle, challenge, deviation from the plan	Individual patient/practitioner level	To increase implementation/ability of staff to deliver intervention successfully
BA SOP was revised to create tailored sessions (timing, frequency, referrals, resources, etc.).	The BA SOP originally was not tailored to each patient’s personal preference for timing, frequency, etc.	Tailoring/rewording/refining	post-implementation	Reactive: Unplanned often in response to an obstacle, challenge, deviation from the plan	Individual patient/practitioner level	To increase implementation/ability of staff to deliver intervention successfully
BA SOP was revised to include suggestions, referrals, and resources for sleep, pain, and alternate relaxation techniques during and after the intervention time period.	The BA SOP did not originally have additional suggestions and techniques.	Adding elements	Post-implementation	Reactive: Unplanned often in response to an obstacle, challenge, deviation from the plan	Individual patient/practitioner level	To make intervention more aligned with organization goals
MO SOP was revised to encourage patients to self-advocate and empower themselves to communicate with their prescribers to implement medication changes.	The MO SOP did not originally include guidelines to encourage self-advocacy.	Adding elements	Post-implementation	Reactive: Unplanned often in response to an obstacle, challenge, deviation from the plan	Target intervention group level	To increase effectiveness
BA was tailored for older surgical patients and their specific goals and activities preoperatively and postoperatively (including surgery recovery goals from surgical team).	BA was originally not tailored for different types of surgeries and types of older patient (e.g., retired vs semiretired, family vs. no family).	Integrating intervention into another framework	Post-implementation	Planned: Part of the plan to modify to maximize fit and implementation success	Individual patient/practitioner level	To make intervention more aligned with organization goals
BA SOP was revised to include motivational interviewing techniques to encourage patients who have more resistance to changing their behavior.	The BA SOP did not originally use motivational interviewing techniques.	Adding elements	Post-implementation	Reactive: Unplanned often in response to an obstacle, challenge, deviation from the plan	Individual patient/practitioner level	To increase implementation/ability of staff to deliver intervention successfully
BA documentation forms were reduced in detail and wellness partners were encouraged to reinforce activities in addition to suggesting new ones. Wellness partners were also encouraged to suggest flexible methods of activity documentation (e.g., journaling), and emphasized meeting the patient where they were, not forcing anything upon them.	Previously, wellness partners were encouraged to keep scheduling new activities and goals, without reinforcement. Furthermore, documentation forms were mandatory to the intervention bundle.	Loosening structure	Post-implementation	Reactive: Unplanned often in response to an obstacle, challenge, deviation from the plan	Individual patient/practitioner level	To increase effectiveness
Intervention bundle was renamed to "surgical wellness program" across all intervention documents and research documents	The original intervention bundle was called the “perioperative mental health bundle.”	Tailoring/rewording/refining	Post-implementation	Reactive: Unplanned often in response to an obstacle, challenge, deviation from the plan	Individual patient/practitioner level	To increase reach, participation, access
The activity tracking form was modified to reflect the granularity as defined by the patient	The original activity tracking form was very detailed and required patients to track all their activities	Tailoring/rewording/refining	Post-implementation	Reactive: Unplanned often in response to an obstacle, challenge, deviation from the plan	Individual patient/practitioner level	To increase implementation/ability of staff to deliver intervention successfully
MO SOP was revised to have the pharmacy team lead the MO component – review medications and optimize the targeted medications	Wellness partners originally reviewed medications and provided recommendations	Tailoring/rewording/refining	Post-implementation	Reactive: Unplanned often in response to an obstacle, challenge, deviation from the plan	Target intervention group level	To increase effectiveness
Screening procedure was revised to include a narrative showing that studies indicated BA was effective for anxiety, depression, and general well-being, followed by an explanation of the surgical wellness program.	Previous screening procedures focused heavily on mental health screening, which was stigmatized by patients.	Adding elements	Post-implementation	Reactive: Unplanned often in response to an obstacle, challenge, deviation from the plan	Individual patient/practitioner level	To increase reach, participation, access
Consent language was revised to include a description of what to expect from the surgical wellness program, omitting language about anxiety and depression to avoid stigma.	Previous consent language was complex and vague, which meant that patients did not understand the intervention bundle prior to participation.	Tailoring/rewording/refining	Post-implementation	Reactive: Unplanned often in response to an obstacle, challenge, deviation from the plan	Individual patient/practitioner level	To increase reach, participation, access
Both control and intervention groups in the future RCT will receive resources for mindfulness, relaxation, stress reduction, daily routines, sleep hygiene, activity rest cycle, brain training, and social activities.	Originally, the control group would only receive usual care.	Adding elements	Post-implementation	Planned: Part of the plan to modify to maximize fit and implementation success	Individual patient/practitioner level	To make intervention more aligned with organization goals

**Table 7:** Contextual adaptations.

Adaptations	Original protocol	What was adapted	When adaptation occurred	Planned or reactive	At what level of delivery	Intent of adaptation
MO SOP was revised to involve patients in decision-making and to assign wellness partners with documentation responsibilities, including REDCap forms on medication changes.	The original MO SOP did not factor patients into the decisions that wellness partners made during sessions.	Adding elements	Pre-implementation	Planned: Part of the plan to modify to maximize fit and implementation success	Target intervention group level	To increase reach, participation, access
Sessions were conducted in-person 1:1 informally at first and then over the phone/Zoom following the first session.	Sessions were conducted in accordance with patient preference.	Tailoring/rewording/refining	Pre-implementation	Planned: Part of the plan to modify to maximize fit and implementation success	Target intervention group level	To increase reach, participation, access
Wellness partners used a medication management algorithm in addition to receiving supervision from pharmacists and a geriatric psychiatrist.	Wellness partners originally did MO themselves, in consultation with pharmacists and a geriatric psychiatrist.	Adding elements	Pre-implementation	Planned: Part of the plan to modify to maximize fit and implementation success	Target intervention group level	To increase implementation/ability of staff to deliver intervention successfully
MO and BA sessions were scheduled to be biweekly or weekly for a total of 8–12 sessions. Additional sessions were added if necessary or if goals were not met.	Previously, there was no number of sessions or frequency set – wellness partners were expected to schedule them based on each patient’s individual preferences and availability.	Tailoring/rewording/refining	Pre-implementation	Planned: Part of the plan to modify to maximize fit and implementation success	Individual patient/practitioner level	To increase effectiveness
BA session documentation forms were different for Sessions 1, 2, 3, 4–9, and 10.	Originally, forms were different for Sessions 1, 2, 3–9, and 10.	Tailoring/rewording/refining	Pre-implementation	Planned: Part of the plan to modify to maximize fit and implementation success	Individual patient/practitioner level	To increase implementation/ability of staff to deliver intervention successfully
2–4 BA sessions were conducted preoperatively if possible, ideally starting 30 days prior to surgery and ending sessions 90 days after surgery.	Originally, there was no formal schedule or split between pre-operative and postoperative sessions.	Adjusting the order of intervention components	Pre-implementation	Planned: Part of the plan to modify to maximize fit and implementation success	Individual patient/practitioner level	To increase reach, participation, access
Patients were contacted virtually up to 3 times for intervention sessions and follow-up before wellness partners reached out via mail.	Patients were contacted over email or by phone indefinitely.	Lengthening/extending (pacing/timing)	Post-implementation	Reactive: Unplanned often in response to an obstacle, challenge, deviation from the plan	Individual patient/practitioner level	To increase reach, participation, access
6 pharmacy students assisted wellness partners with MO (with supervision from pharmacists).	Originally, pharmacy students were not included in the study or intervention bundle.	Adding elements	Post-implementation	Reactive: Unplanned often in response to an obstacle, challenge, deviation from the plan	Target intervention group level	To increase implementation/ability of staff to deliver intervention successfully
MO SOP was revised to reflect medication data collection between first session and all other sessions.	The same type of medication data was originally collected at each session, causing some redundancy.	Tailoring/rewording/refining	Post-implementation	Reactive: Unplanned often in response to an obstacle, challenge, deviation from the plan	Target intervention group level	To increase implementation/ability of staff to deliver intervention successfully
MO SOP was revised to reflect the pharmacy team's roles and responsibilities.	Originally, the pharmacy team’s roles and responsibilities did not extend to MO.	Tailoring/rewording/refining	Post-implementation	Reactive: Unplanned often in response to an obstacle, challenge, deviation from the plan	Target intervention group level	To increase implementation/ability of staff to deliver intervention successfully
BA and MO began in the same session.	Originally, BA began one session after MO.	Adjusting the order of intervention components	Post-implementation	Planned: Part of the plan to modify to maximize fit and implementation success	Individual patient/practitioner level	To increase implementation/ability of staff to deliver intervention successfully
Patients were reminded of their goals and about activities that made them feel good or mattered to them. They were also reminded that the goals of the study were to support overall surgical recovery, not just mental health.	Originally, BA SOP language emphasized mental health improvement and recovery, rather than overall surgical recovery.	Adding elements	Post-implementation	Reactive: Unplanned often in response to an obstacle, challenge, deviation from the plan	Individual patient/practitioner level	To increase implementation/ability of staff to deliver intervention successfully
Patients were offered opportunities to reach out to their wellness partners as needed within the 3-month intervention period, and were encouraged to check in monthly.	Originally, there was no guideline for patients to keep in touch with their wellness partners.	Adding elements	Post-implementation	Reactive: Unplanned often in response to an obstacle, challenge, deviation from the plan	Individual patient/practitioner level	To increase reach, participation, access
Exclusion criteria was modified to exclude joint replacement surgery patients, patients with immediate suicidal ideation, and rescheduled surgical patients who have canceled or postponed surgeries within the past 3 months following enrollment into the study; inclusion criteria were modified to include patients 60 years of age and older.	The study originally included joint replacement patients, patients with suicidal ideation, and rescheduled surgical patients. The study originally excluded patients under 65 years of age.	Removing/skipping elements	Post-implementation	Reactive: Unplanned often in response to an obstacle, challenge, deviation from the plan	Individual patient/practitioner level	To increase reach, participation, access
Follow-up assessment surveys were optionally emailed to patients.	Follow-up assessment surveys were originally only administered via phone call.	Tailoring/rewording/refining	Post-implementation	Reactive: Unplanned often in response to an obstacle, challenge, deviation from the plan	Individual patient/practitioner level	To increase reach, participation, access
Employment status was collected during enrollment.	Originally, employment status was not collected.	Adding elements	Post-implementation	Reactive: Unplanned often in response to an obstacle, challenge, deviation from the plan	Individual patient/practitioner level	To increase reach, participation, access
Auto-generated calendars with follow-ups (throughout study and at end of study) were suggested for future RCT use.	Originally, wellness partners notified the research coordinator of patient progress via email.	Substituting components	Post-implementation	Planned: Part of the plan to modify to maximize fit and implementation success	Individual patient/practitioner level	To increase implementation/ability of staff to deliver intervention successfully
Data collection was revised to gather all medication lists from Epic and confirm them in each session to ensure in the future that the research coordinator is blinded.	The study team originally planned that data would be collected by the research coordinator, who would then know which patients were in each arm of the study.	Tailoring/rewording/refining	Post-implementation	Planned: Part of the plan to modify to maximize fit and implementation success	Individual patient/practitioner level	To increase effectiveness
Intervention sessions could be scheduled differently based on type of surgery – orthopedic patients typically scheduled their surgeries 3 + months in advance and had more time for pre-operative sessions. In contrast, oncologic patients scheduled their surgeries about 2 weeks in advance, and cardiac patients scheduled their surgeries about 2–3 days in advance, leaving little room for pre-operative sessions.	Originally, there was no plan of scheduling sessions differently based on type of surgery.	Adjusting the order of intervention components	Post-implementation	Reactive: Unplanned often in response to an obstacle, challenge, deviation from the plan	Target intervention group level	To increase reach, participation, access
Caregivers were not included in the intervention bundle.	Originally, caregiver involvement was optional and encouraged.	Removing/skipping elements	Post-implementation	Planned: Part of the plan to modify to maximize fit and implementation success	Individual patient/practitioner level	To increase effectiveness
Wellness partners were instructed to deliver the intervention bundle with elements of compassion and patient-sensitivity.	Originally, wellness partners did not intentionally incorporate elements of compassion into their sessions.	Adding elements	Post-implementation			

**Table 8:** Training and evaluation adaptations.

Adaptations	Original protocol	What was adapted	When adaptation occurred	Planned or reactive	At what level of delivery	Intent of adaptation
Wellness partners were trained based on previous work by Puspitasari et al. [47].	Original protocol did not specify wellness partner training	Adding elements	Pre-implementation	Planned: Part of the plan to modify to maximize fit and implementation success	Individual patient/practitioner level	To increase implementation/ability of staff to deliver intervention successfully
Wellness partners were retrained throughout the intervention implementation.	Original protocol did not utilize retraining sessions for wellness partners	Adding elements	Post-implementation	Planned: Part of the plan to modify to maximize fit and implementation success	Individual patient/practitioner level	To increase implementation/ability of staff to deliver intervention successfully
Pharmacy students were trained on how to support wellness partners during MO.	Original protocol did not train pharmacy students to aid in MO	Adding elements	Post-implementation	Reactive: Unplanned often in response to an obstacle, challenge, deviation from the plan	Individual patient/practitioner level	To increase implementation/ability of staff to deliver intervention successfully
Data collection was simplified, including revision of suicide risk, alcohol consumption, opioid, falls, and medication questions.	The research coordinator used the Behavioral Activation for Depression Scale – Short Form (BADS-SF) and the Veterans RAND – 12 (VR-12) to measure target engagement and quality of life.	Loosening structure	Post-implementation	Reactive: Unplanned often in response to an obstacle, challenge, deviation from the plan	Individual patient/practitioner level	To increase implementation/ability of staff to deliver intervention successfully
Data on hospital readmissions and follow-ups were collected.	Originally, data on hospital readmissions and follow-ups were not collected.	Adding elements	Post-implementation	Planned: Part of the plan to modify to maximize fit and implementation success	Individual patient/practitioner level	To increase reach, participation, access
Only 1-month and 3-month follow-ups were collected.	Originally, 1-month, 2-month, and 3-month follow-ups were collected.	Removing/skipping elements	Post-implementation	Reactive: Unplanned often in response to an obstacle, challenge, deviation from the plan	Target intervention group level	To increase reach, participation, access
REDCap session documentation forms were revised to include a general emotional health question.	Originally, REDCap had a question that assumed that the patient had depression and anxiety and forced patients to provide ratings.	Adding elements	Post-implementation	Reactive: Unplanned often in response to an obstacle, challenge, deviation from the plan	Individual patient/practitioner level	To increase implementation/ability of staff to deliver intervention successfully
Future RCTs will use chart-del methods to obtain delirium assessments.	The current study used in-person CAM assessments.	Substituting components	Post-implementation	Planned: Part of the plan to modify to maximize fit and implementation success	Individual patient/practitioner level	To increase implementation/ability of staff to deliver intervention successfully

## Abbreviations

PMH: Perioperative mental health
MOD: Medication optimization and deprescription
MO: Medication optimization
BA: Behavioral activation
IAB: Internal advisory board
FRAME: Framework for Reporting Adaptations and Modifications – Expanded
SOP: Standard operating procedure
